# Patterning Techniques in Coplanar Micro/Nano Capacitive Sensors

**DOI:** 10.3390/mi14112034

**Published:** 2023-10-31

**Authors:** Seokwon Joo, Jung Yeon Han, Soonmin Seo, Ju-Hyung Kim

**Affiliations:** 1 Department of Chemical Engineering and Department of Energy Systems Research, Ajou University, Suwon 16499, Republic of Korea; 2 Department of Bionano Technology, Gachon University, Seongnam 13120, Republic of Korea; jhan@gachon.ac.kr

**Keywords:** parallel-plate capacitive sensors, coplanar-type capacitive sensors, patterning techniques, inkjet printing, screen printing, laser patterning, soft lithography, 3D sensors

## Abstract

Rapid technological advancements have led to increased demands for sensors. Hence, high performance suitable for next-generation technology is required. As sensing technology has numerous applications, various materials and patterning methods are used for sensor fabrication. This affects the characteristics and performance of sensors, and research centered specifically on these patterns is necessary for high integration and high performance of these devices. In this paper, we review the patterning techniques used in recently reported sensors, specifically the most widely used capacitive sensors, and their impact on sensor performance. Moreover, we introduce a method for increasing sensor performance through three-dimensional (3D) structures.

## 1. Introduction

Sensors have emerged as core components of specific analytes or motion detection as well as next-generation technologies such as machine learning, big data, and artificial intelligence [1,2,3]. Various types of sensors exist, depending on the driving mechanism, such as resistive, piezoelectric, triboelectric, and capacitive, and the appropriate type employed depends on the application. Capacitive sensors are one of the most used for sensing mechanical movements (e.g., touch, pressure, and tilt), chemical reactions (e.g., changes in pH, heat, and electron flow), and biological reactions (e.g., those involving a combination of enzymes, DNA, and antibodies) due to their high sensitivity and resolution as well as their simple structure and low power consumption [4,5,6]. In addition, they are actively applied to commercial electronic products such as touch panels, robots, and wearable devices (e.g., E-skins and prosthetic devices) owing to their low sensitivity to temperature and drift [7,8,9]. Although they possibly suffer from time-dependent parasitic capacitance issues that can arise from interaction with external environments, they can be mitigated through sealing techniques or digitized signals [10]. This review provides an overview of the types of capacitive sensors and patterning techniques required for sensor fabrication, specifically focusing on coplanar sensors. Furthermore, a capacitive sensor with a 3D structure is introduced.

**Figure 1:**
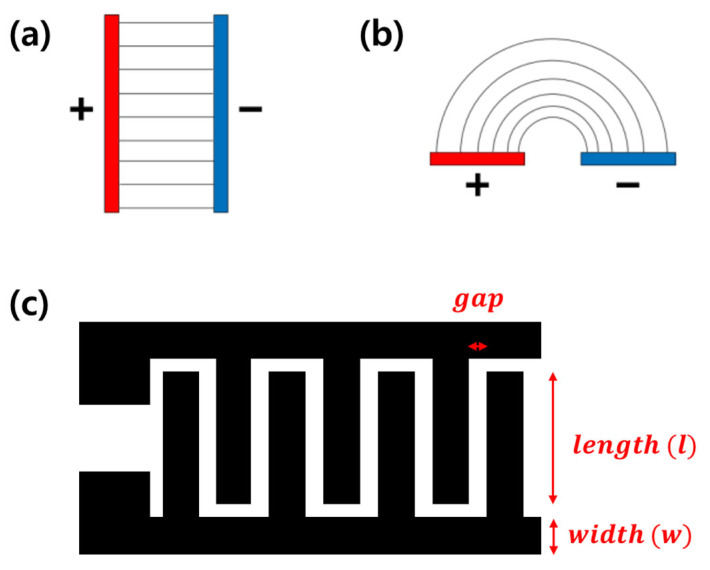
(**a**) Perpendicular-type and (**b**) horizontal-type capacitive sensors [11]. (**c**) Scheme of the interdigitated pattern.

Capacitive sensors detect changes in the capacitance between two electrodes in response to external stimuli. From the perspective of the electrode structures, sensors can be classified into two types: perpendicular and horizontal (Figure 1).

In a typical perpendicular-type capacitive sensor, also called a sandwich or parallel-plate structure, two flat-plate electrodes facing each other are separated by an insulator. The capacitance formula for this structure is as follows [4]:

(1) $$C=\varepsilon \frac{A}{d}$$

where ε is the relative dielectric constant of the dielectric layer, A is the area of the electrodes facing each other, and d is the distance between the two electrodes. Figure 1a shows the basic structure of a perpendicular capacitive sensor. This sensor is usually used as a mechanical sensor (e.g., for sensing pressure and strain) due to its structural characteristics, and the capacitance changes depending on the thickness of the dielectric material under pressure. For applications in structures such as touch panels, both electrodes are usually lined and crossed into a matrix pattern [12,13,14]. However, owing to the poor sensitivity of this sensor type, the electrodes are frequently patterned to improve the sensor’s performance. These electrode surfaces contain artificial patterns (e.g., pyramids, cylinders, and hemispheres), including biomimetic patterns (e.g., lotus leaf surfaces) [15,16,17], simply leading to an increase in roughness and specific surface area.

The horizontal-type sensor, also called a coplanar-type sensor, contains two electrodes placed on a substrate, unlike with the parallel-plate type (Figure 1b). Due to its structural characteristics, it is widely used for physical measurements as well as chemical and biological detection through binding receptors to electrodes, from simple structures, such as triangles and squares, to efficient structures, such as swirls and combs [18,19,20,21,22,23]. Among them, the comb-shaped structure, also called an interdigitated (ID) pattern, is the most used because it is a simple structure with maximal efficiency in the same area (Figure 1c) [24]. In a sensor based on an ID pattern, the sensitivity and signal strength are proportional to the number and length of electrode fingers [25]. The equation for total capacitance in the ID pattern, *C_s_*, is as follows:

(2) $$C_{s}=n\cdot l\cdot \left(C_{IDE}+C_{SUB}+C_{SEN}\right)$$

where n is the number of electrode fingers, l is the length of overlapping fingers, $C_{IDE}$ is the transmission-line capacitance, $C_{SUB}$ is the capacitance in the presence of a substrate, and $C_{SEN}$ is capacitance in the sensing area.

## 2. Surface Patterning to Improve Parallel-Plate Capacitive Sensors

**Figure 2:**
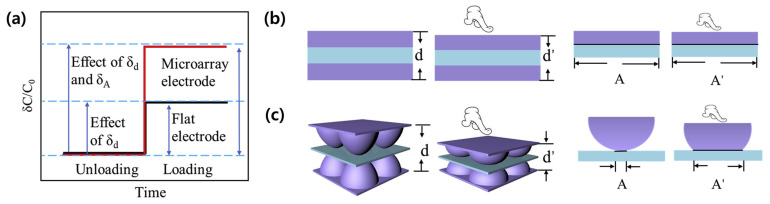
(**a**) The effect of A and d on capacitance change where A (A′) is the area of the electrodes facing each other, and d (d′) is the distance between the two electrodes. Schematics showing change of A and d from the external stimulus in (**b**) basic flat structure and (**c**) patterned electrode structure. Reproduced with permission from [26], [Nano Energy]; published by Elsevier, 2020.

In pressure or touch sensors, which are the main applications, the parallel plate type spreads stress evenly over the sensing area when subjected to pressure, and performance deteriorates due to small changes in A and d (Equation (1)). Therefore, the performance of the sensor can be improved by micro/nanopatterning on the surface of electrodes or dielectrics to increase the change of those factors under external stimulus, as shown in Figure 2. In this chapter, we review recent reports on various patterns and patterning methods that improve the performance of planar-type capacitive sensors.

### 2.1. Convex Patterns

**Figure 3:**
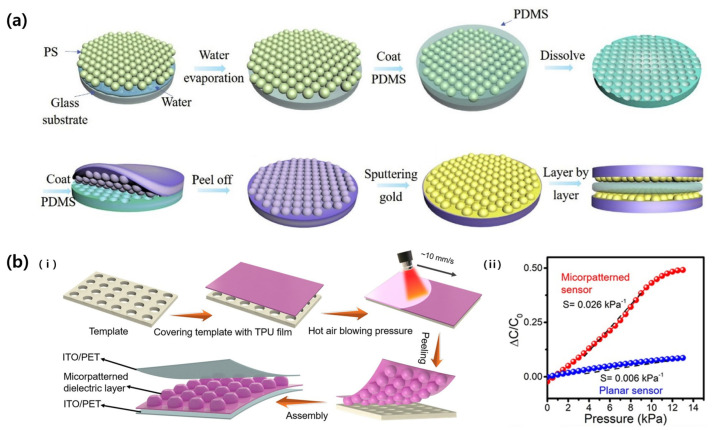
(**a**) Schematics the fabrication process of convex hemisphere electrode based parallel capacitive pressure sensor [26]. (**b**): (i) The fabrication schemes of quasi-hemispherical microarray dielectric layered capacitive sensor. (ii) The graph showing the difference in sensitivity of pressure sensors composed of patterned and non-patterned dielectrics. Reproduced with permission from [27], [Composites Science and Technology]; published by Elsevier, 2023.

Xiong et al. (2020) improved the performance of a pressure sensor by patterning a uniform array of convex hemispheres on the surface of the electrode using soft lithography technology [26]. First, polystyrene microbeads with a 2 μm diameter were arranged through self-assembly, and then poly(dimethylsiloxane) (PDMS) was poured and cured to produce a concave mold (Figure 3a). PDMS was poured again into this concave mold to obtain a convex hemisphere-arranged PDMS mold, and then coated with Au through sputtering. The pressure sensor with the convex microarray electrode could be applied to motion monitoring with high sensitivity (30.2 kPa^−1^), fast response time (25 ms), and low limit of detection (LOD) (0.7 Pa).

Zhang et al. (2023) also increase the performance of a pressure sensor by patterning an array of quasi-hemispherical micropatterns [27]. To prepare, a 30 μm thick polyurethane film was placed on a stainless steel mold with an array of circular holes, and hot air was blown on it (Figure 3b(i)). The temperature of the polyurethane surface rose to 150 °C at this time, and quasi-hemispheres with a diameter of 100 μm were arranged at 100 μm intervals onto the film after the process. The polyurethane film with a patterned surface served as a dielectric between the ITO electrodes, and the fabricated capacitive sensor showed a sensitivity of 0.026 kPa^−1^ and a response time of 99 ms, which was better than that with a flat polyurethane (Figure 3b(ii)).

**Figure 4:**
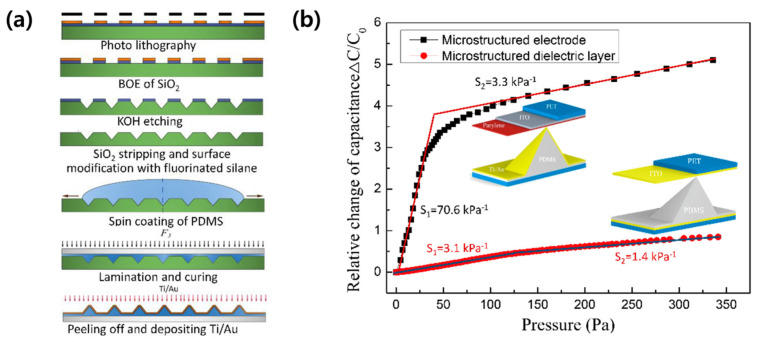
(**a**) The fabrication steps of pyramidal structure pressure sensor. (**b**) Differences in sensitivity depending on the structure [28].

Li et al. (2020) applied a pyramid structure to the electrode through a series of lithography processes to improve the performance of a pressure sensor [28]. After fabricating a pyramid pit array on a SiO_2_ substrate through photo lithography and etching processes, PDMS was poured and cured (Figure 4a). Ti/Au was deposited on a pyramidal microstructure-arranged PDMS layer and used as electrodes, and the pressure sensor, including it, had a sensitivity of 70.6 kPa^−1^ and an LOD of 1 Pa. As shown in Figure 4b, the capacitive sensor of the model using a thin dielectric layer and electrodes deposited on an elastic pyramid-shaped microstructure showed improved sensitivity over 20 times in the same dimension compared to the model using a pyramid-shaped dielectric layer on a flat electrode.

### 2.2. Pillar Patterns

**Figure 5:**
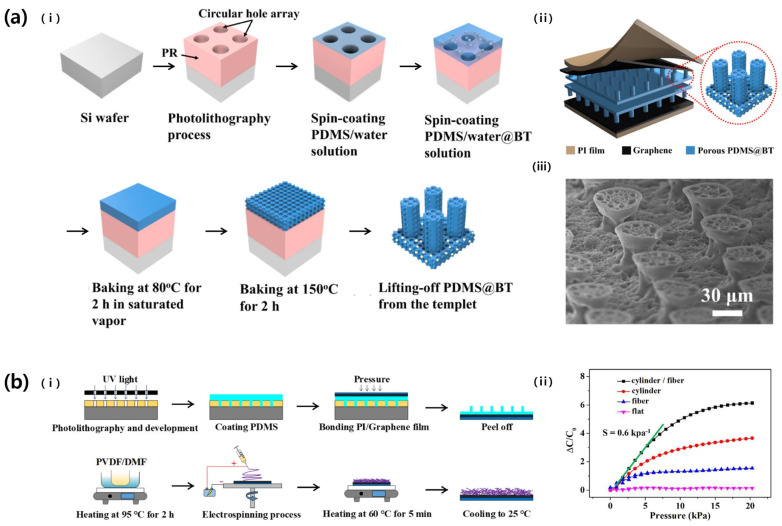
(**a**): (i) Fabrication processes of porous micropillar dielectric layer. (ii) The structure of the capacitive pressure sensor. (iii) SEM image of the porous micropillar structure. Reproduced with permission from [29], [Ceramics International]; published by Elsevier, 2022. (**b**): (i) The fabrication schematics of microcylinder/electrospun fiber dual dielectric layer. (ii) Relative sensitivity comparison depending on the dielectric type. Reproduced with permission from [30], [Organic Electronics]; published by Elsevier, 2021.

In 2022, Yang et al. improved the performance of a pressure sensor by patterning the dielectric layer with a porous micropillar (Figure 5a) [29]. For this purpose, a mold for the micro-pillar was manufactured through photolithography. PDMS, mixed with water and barium titanate particles, was used as the dielectric material. After the structure was formed, evaporation of water left pores in the micropillars, reducing the hardness of the dielectric layer, and the particles enhanced the relative permittivity. The fabricated dielectric layer was applied to a pressure sensor as a double-layer sandwich structure between graphene electrodes, and the sensor’s sensitivity was 7.847 kPa^−1^, LOD was 0.21 Pa, and response time was 20 ms.

Similarly, Lin et al. (2021) increased the performance of a pressure sensor by patterning the dielectric layer into microcylinders using a mold fabricated through photolithography (Figure 5b) [30]. At this time, the diameter and height of the cylinder were 25 μm and 55 μm, respectively. In addition, PVDF fibers with a diameter of 0.8 μm produced by electrospinning were used together in the dielectric layer to further increase performance. The sensor has an improved sensitivity of 0.6 kPa^−1^, a response time of 25 ms, and an LOD of 0.065 Pa.

### 2.3. Biomimetics

Zhao et al. (2023) fabricated a pressure sensor with improved performance using the microstructural pattern of rose petals [31]. The surface of a rose petal was simply copied by pouring and curing PDMS, followed by being sputtered with gold and used as an electrode for a pressure sensor. The pressure sensor fabricated with microstructure electrodes and an AgNWs/PVDF dielectric layer showed a sensitivity of 0.32 kPa^−1^. The hierarchical structure of the petal surface not only increases sensor performance but also causes strong surface adhesion due to the Cassie impregnating state, which can solve wetting problems when applied to e-skin.

**Figure 6:**
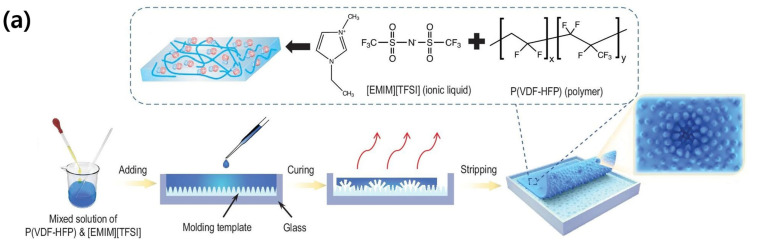

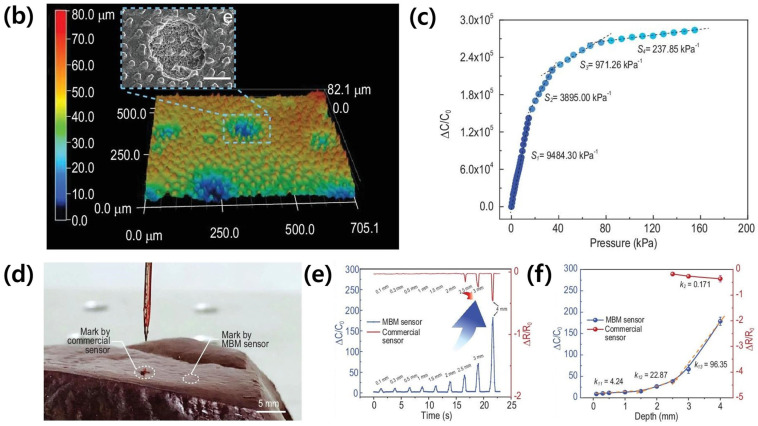
(**a**) The fabrication diagrams of pollen-inspired ionic gel based high-sensitive pressure sensor. (**b**) The 3D topography of the micro-cone surface structured PDMS template. (**c**) Capacitance change upon a pressure. (**d**) Penetration marks when identifying needle-tissue contact using commercial and MBM sensors. (**e**) Real-time monitoring of needle penetration comparison with commercial sensors. (**f**) The sensitivity upon a penetration depth of the MBM and commercial sensor [32].

Wei was inspired by the multistage bionic microstructures (MBMs) of the *M. aquaticum* pollen surface to implement an ultra-sensitive e-skin for the tactile sensor of a surgical robot (Figure 6) [32]. Since the surface of the *C. zebrine* leaf is similar to the micro-cone structure of the pollen surface, PDMS was used to copy the structure. Afterwards, P(VDF-HFP)/[EMIM][TFSI], an ionic gel solution, was poured onto the PDMS template to fabricate a microstructure surface ionic gel, which was used as a dielectric layer for a pressure sensor. The pressure sensor showed a very high sensitivity of 9484.3 kPa^−1^, LOD of 0.12 Pa, and a response time of 24 ms. Using this ultra-sensitive property, it could be applied to robot-assisted invasive surgical incisions to provide tactile information.

**Figure 7:**
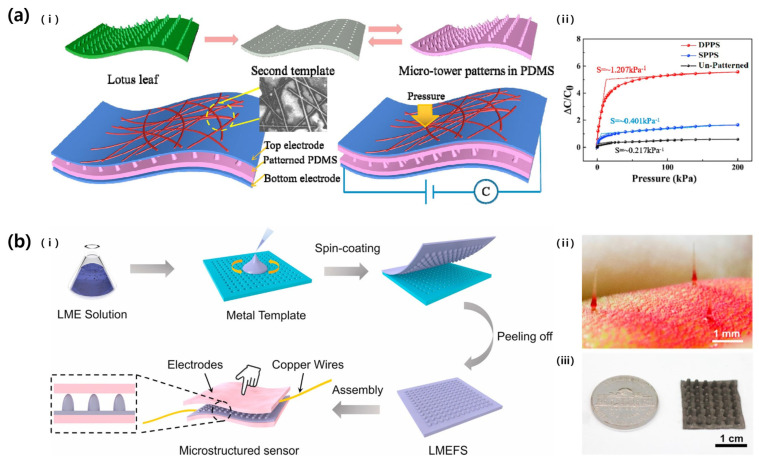
(**a**): (i) Schematic of the fabrication steps and structure of the lotus leaf-inspired pressure sensor. Structures showing a single-layered PDMS-based pressure sensor (SPPS) and double-layered PDMS-based pressure sensor (DPPS). (ii) Graph showing the sensitivity depending on the structure of the dielectric layer. Reproduced with permission from [33], [Current Applied Physics]; published by Elsevier, 2021. (**b**): (i) Schematic diagram showing the fabrication process of LMEFS pressure sensor. Photographs of the (ii) pillar structure in Venus flytrap and (iii) LMEFS. Reproduced with permission from [34] [Composites Science and Technology]; published by Elsevier, 2021.

Zhao et al. (2021) also reported a pressure sensor fabrication inspired by the surface of a lotus leaf (Figure 7a) [33]. The microtower structure of the lotus leaf was copied twice through PDMS pour-curing processes. The low density of the lotus leaf pattern causes a large change in dielectric layer thickness even at low pressure, increasing sensitivity. The sensitivity of the manufactured pressure sensor was 1.207 kPa^−1^, the LOD was 0.02 kPa, and the response time was 61.6 ms.

Meanwhile, Zhang et al. (2021) enhanced the performance of the pressure sensor by utilizing a pillar structure that mimics the trigger hair of the Venus flytrap (Figure 7b) [34]. To prepare the pattern, a laser was irradiated on a copper plate, and the pattern was engraved. The pattern was a hole with a diameter of 2 mm at the top, a diameter of 1 mm at the bottom, and a height of 3 mm. Then a mixture of Galinstan (liquid metal) and Ecoflex 00-30 (elastomer; Smooth-on, Macungie, PA, USA) was spin-coated on this template to create a liquid metal (LM) elastomer with a flytrap-inspired structure (LMEFS), which was used as a dielectric layer for a pressure sensor. LM particles mixed into the dielectric layer increased the dielectric constant of the dielectric layer without increasing the modulus like other fillers (carbon-based materials, metal particles). As a result, the sensor had a sensitivity of 1.061 kPa^−1^, a hysteresis of 13.8%, a response time of 45 ms, and a large initial capacitance of 2.57 pF (at 40% LM content, 2.4 times compared to 0%).

## 3. Printing Techniques for Fabrication of Coplanar-Type Capacitive Sensors

**Figure 8:**
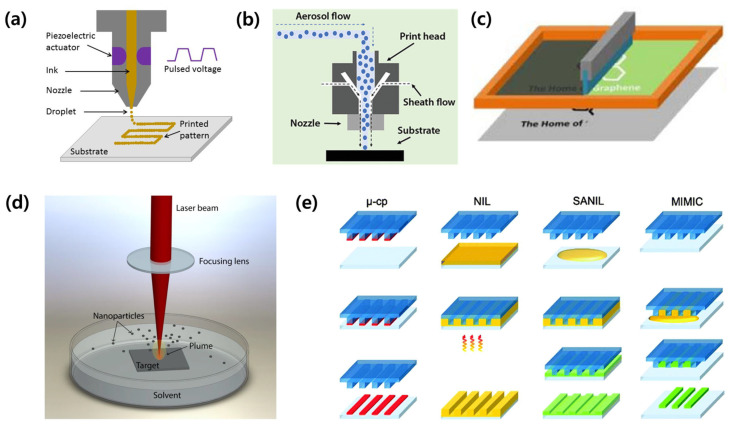
Schematics of the patterning methods used to fabricate coplanar-type capacitive sensors. (**a**) Inkjet printing [36]. (**b**) Aerosol jet printing. Reproduced with permission from [37], [Advanced Materials Technologies]; published by John Wiley and Sons, 2023. (**c**) Screen printing. Reproduced with permission from [38], [Applied Materials]; published by American Chemical Society, 2019. (**d**) Laser printing [39]. (**e**) Soft lithography [40].

Various patterning methods can be employed to establish patterns in sensors, and these should be selected in consideration of the characteristics of the materials and substrates (Figure 8) [35]. This section introduces patterning technologies related to the coplanar type (i.e., inkjet printing, aerosol jet printing, screen printing, laser patterning, and soft lithography), which are widely used for the fabrication of capacitive sensors.

### 3.1. Inkjet Printing

Inkjet printing is a widespread technology that directly sprays a target material onto a substrate through a nozzle. This digitally controlled technology is used to perform direct printing that does not require a mask, and the substrate has few restrictions. In addition, many advantages (i.e., high accuracy and repeatability, low costs, and can perform large-area printing) lead this method to be utilized in various fields such as biomedical, energy storage, and electronics [41,42,43]. However, it has issues with throughput, resolution, and positioning accuracy. Moreover, nozzles are clogged when the ink concentration is high, and a coffee-ring effect appears when there is a non-uniform distribution in the pattern; thus, it is crucial to optimize the ink [44,45,46].

**Figure 9:**
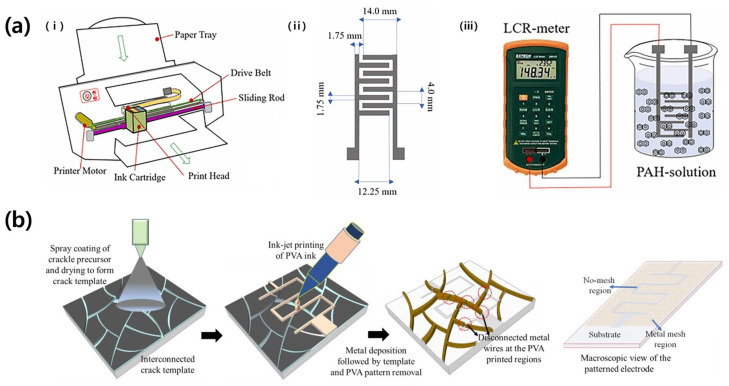
(**a**): (i) Scheme of inkjet printing process. (ii) Design of ID pattern printed via inkjet printing. (iii) Measuring scheme of PAH sensor. Reproduced with permission from [47], [Materials Today Communications]; published by Elsevier, 2023. (**b**) Fabrication schematics of patterned mesh ID electrode. Reproduced with permission from [48], [Materials Letters]; published by Elsevier, 2023.

Pillai et al. (2023) reported a capacitive sensor capable of detecting polycyclic aromatic hydrocarbons (PAHs), environmental toxicants present in aquatic environments (Figure 9a) [47]. Silver nano-ink was used as the sensor electrode material, and it was printed on polyethylene terephthalate (PET) using inkjet printing technology. The surface energy and roughness of the substrate were increased using O_2_ plasma treatment to improve the adhesion of the ink. The electrode was designed according to the ID pattern with a total area of 316.61 mm^2^, the width and length of the fingers were 1.75 mm and 33.3 mm, respectively, and the total number of electrode fingers was eight. The LOD of the sensor for PAHs in aqueous media was 0.05 ng/mL, showing high sensitivity.

In 2022, Mondal et al. studied a transparent wearable tactile-cum-proximity capacitive sensor by utilizing inkjet printing (Figure 9b) [48]. Thermally deposited aluminum on a PET substrate was used as the electrode for the sensor. After printing polyvinyl alcohol (PVA) in a serpentine pattern using inkjet printing, aluminum was deposited thereon. Subsequently, the PVA was lifted off to fabricate the aluminum into an ID pattern. The pattern was fabricated on a large area of 30 × 30 cm^2^, and the distance between the electrode fingers was 300 μm. The fabricated sensor was able to detect non-touch up to 9 cm away from the sensor, while touch detection had a high sensitivity of 1000%.

### 3.2. Aerosol Jet Printing

Aerosol jet printing is a recently emerging printing method that is widely used in micro manufacturing, such as microfluidic devices, due to its digitized direct writing, non-contact, and high resolution (≈10 µm) [37,49]. Atomized ink through ultrasound, gas mechanics, and spark discharge flows into carrier gases and is sprayed onto the substrate [50,51]. At this time, clogging of the nozzle can be prevented by flowing the sheath gas together [52].

**Figure 10:**
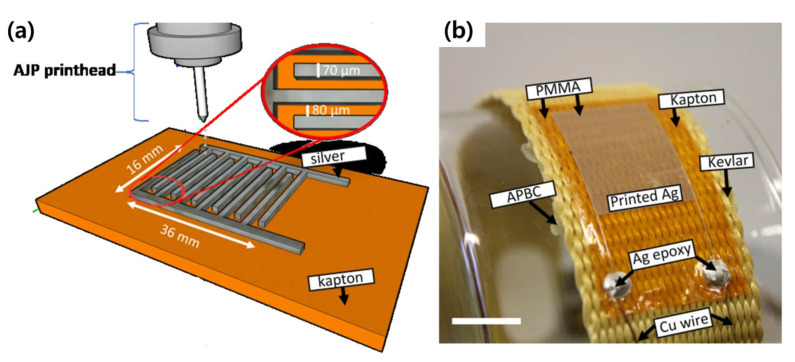
(**a**) Schematic of fabrication process of ID electrode through aerosol jet printing method. (**b**) Optical image of the strain sensor (scale bar represents 1 cm) [53].

Fujimoto et al. (2020) reported the production of a capacitive strain sensor using aerosol jet printing (Figure 10) [53]. Commercial aerosol jet printing was used (Optomec Aerosol Jet 200), and silver nanoparticle ink was pneumatically atomized and sprayed on a polyimide substrate through a 200 µm nozzle. The pattern was designed in an ID shape, and the finger width, length, and spacing were approximately 70 µm, 15 mm, and 80 µm, respectively. The fabricated strain sensor had initial capacitance values ranging from 42 pF to 15 nF and a gauge factor of 5.2.

### 3.3. Screen Printing

Screen printing is used to print a desired pattern using a screen or stencil as a mask. Squeezing is used to pass the ink through the mask and print onto the substrate. It is widely used industrially because of its ability to produce patterns with high resolution and accuracy in a large area at high speed.

**Figure 11:**
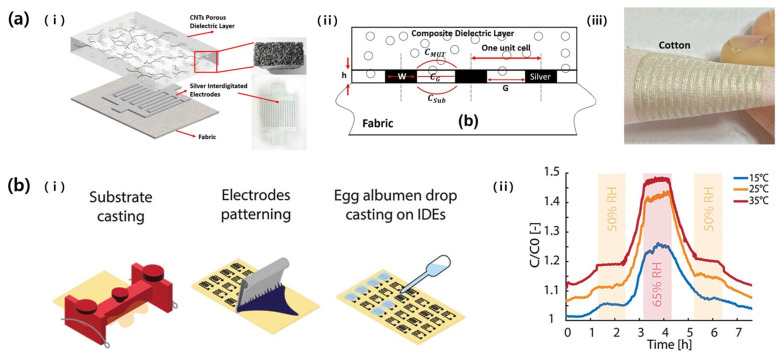
(**a**): (i) Schematics of a capacitive pressure-sensor structure consisting of ID Ag electrodes and an Ecoflex/CNT microporous layer. (ii) Cross-section of the pressure sensor. (iii) Highly flexible sensor fabricated on cotton substrate [54]. (**b**): (i) Schematics of the fabrication steps of the humidity sensor by using screen printing. (ii) The graph of the relative capacitance under the RH from 30 to 70% at different temperatures [55].

Truong et al. (2022) studied a wearable capacitive pressure sensor by using the screen-printing technique (Figure 11a) [54]. Polyester and cotton fabric were used as substrates, and commercially available silver paste (DM-SIP-2001) was printed in the shape of an ID pattern. The width and length of the electrode fingers were 0.57 mm and 15 mm, respectively, and the gap between the fingers was 0.71 mm. Thereafter, a microporous film consisting of Ecoflex and CNT was coated on the electrode to increase the sensitivity of the sensor. As a result, the sensor could measure up to 400 kPa, and the sensitivity ranged from 0.035 (at 400 kPa) to 0.15 KPa^−1^ (at 50 kPa).

Aeby et al. (2023) produced a renewable and biodegradable humidity sensor using a screen printing method [55]. They printed an ID pattern of electrodes using carbon ink containing graphite flakes and carbon black using a commercial screen printer and polyester mesh (Figure 11b). The sensing area was 1 cm^2^, the width and gap of the fingers were 200 µm, the length was 10.1 mm, and the number of fingers was 24. Afterwards, egg albumin was drop-cast on the sensing area, which is sensitive to humidity, increasing the sensor’s response and recovery speed by about 20 times and achieving a sensitivity of 0.011% RH^−1^.

### 3.4. Laser Patterning

Laser ablation, or laser patterning, produces patterns by inducing phenomena such as sintering, evaporation, melting, and solidification. It utilizes thermal energy and irradiates a laser beam on a sample. Owing to high-density energy being exposed to a local area, sample damage, such as cracks, distortion, and burning, may occur. Therefore, optimization of process parameters related to the laser energy density, such as the laser-spot size, power, speed, and frequency, is required [56,57]. It is a preferred patterning technique because it does not require a mask and is highly accurate and productive.

**Figure 12:**
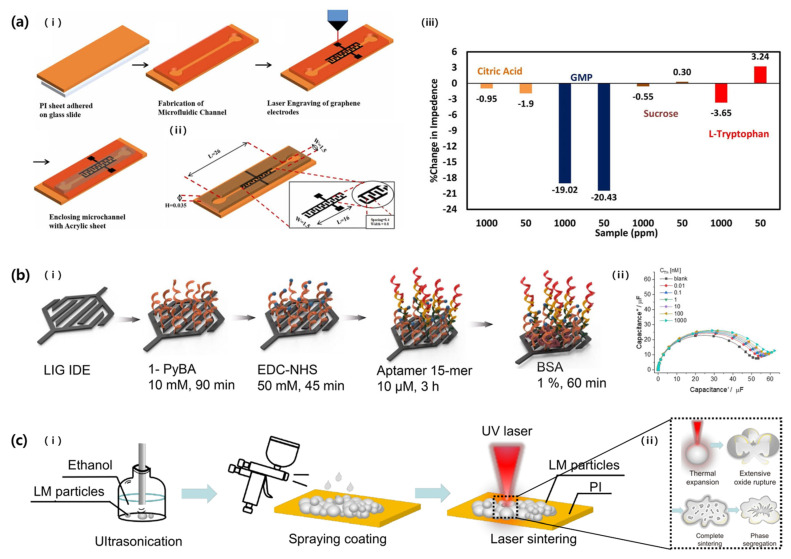
(**a**): (i) Schemes of ID-pattern graphene electrode fabricated via laser irradiation on a polyimide film. (ii) Scheme showing specifications of an ID pattern. (iii) Graph showing percentage change in impedance for different sample concentrations (50, 1000 ppm). Reproduced with permission from [58], [Sensors and Actuators A: Physical]; published by Elsevier, 2022. (**b**): (i) Schematics of modification steps of LIG based biosensor. (ii) Nyquist capacitive plots showing analysis range of the sensor. Reproduced with permission from [59], [Biosensors and Bioelectronics]; published by Elsevier, 2020. (**c**): (i) Schematics of the fabrication processes of LM-based humidity sensor. (ii) Mechanism by which conductive path of LM is formed via laser irradiation [60].

Wagh et al. (2022) explored a microfluidic taste sensor by using laser ablation (Figure 12a) [58]. A polyimide film was used as a substrate, and graphene was partially developed by irradiating a CO_2_ laser (1.95 W, 2.5 cm/s). Laser-induced graphene (LIG) was printed in an ID pattern and used as an electrode. The width of the electrode finger and the gap between the fingers were 0.8 mm and 0.4 mm, respectively. Five analytes, namely sucrose (sweet), citric acid (sour), guanosine monophosphate (umami), sodium chloride (salty), and L-tryptophan (bitter), were prepared at 1–1000 ppm each. Their lower limits were 2.92, 5.205, 2.45, 17.11, and 4.89 µM, respectively.

Yagati et al. (2020) also fabricated LIG into an ID pattern and used it in a biosensor for thrombin detection (Figure 12b) [59]. LIG was patterned with a polyimide substrate as a precursor and an ID pattern with a finger width and gap of 200 µm and 45 µm thickness. The sensor had an analysis range from 0.01 nM to 1000 nM and a low LOD of 0.12 pM due to the high porosity of LIG, and also had excellent repeatability and long-term stability (over 7 days at 4 °C). Furthermore, the sensitivity of the LIG electrode may improve due to changes in the dielectric constant as aptamer antigens are labeled.

In 2023, Cui et al. showed a flexible LM-based humidity sensor using laser ablation (Figure 12c) [60]. After spray-coating the LM onto a polyimide film, a UV laser (6.8 J/cm^2^) was irradiated to rupture the oxide skin of the LM nanoparticles to form a conductive network (resistivity of approximately 0.19 Ω·cm). The conductive path was printed in an ID pattern, and the number of electrode fingers was six. When the relative humidity changed from 30% to 90%, the highest capacitance change value was 142.4%, which was observed when the finger width was 1.5 mm and the length was 11 mm.

### 3.5. Soft Lithography

Soft lithography is a technique used to fabricate structures or patterns using elastomeric stamps (e.g., PDMS or Ecoflex) with a patterned surface. Using elastomeric stamps, various sub-techniques, such as microcontact printing, replica molding, and micro transfer molding, can be applied [61]. Soft lithography is a promising fabrication technology due to its low cost and easy process; however, its repeatability is low, and distortion may occur due to deformation of the elastomeric stamp (i.e., pairing, sagging, swelling, or shrinking) [62].

**Figure 13:**
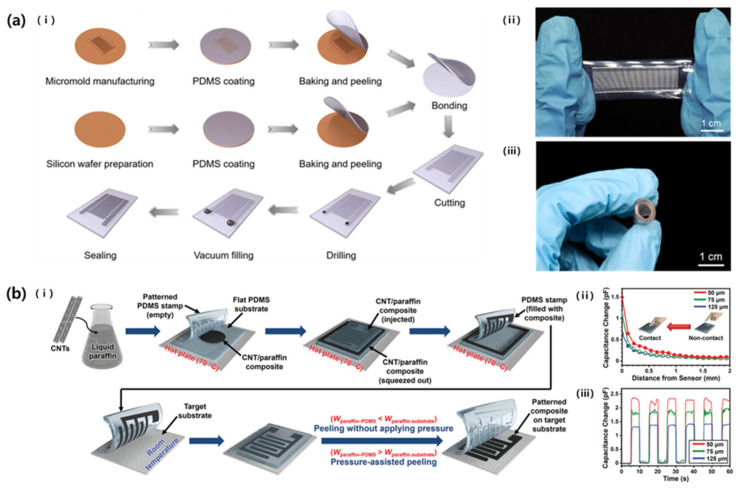
(**a**): (i) Schematics of fabrication process of liquid-metal-based capacitive strain sensor via soft lithography. Photographs showing the (ii) stretchability and (iii) bendability of the flexible sensor [63]. (**b**): (i) Schematics showing the fabrication steps of CNT composite pattern by using intaglio contact printing method. The graph showing sensor (ii) resolution and (iii) sensitivity depending on the pattern width. Reproduced with permission from [64], [Small]; published by Wiley-VCH GmbH, 2022.

Zhang et al. (2022) studied an LM-based capacitive strain sensor, which was fabricated via soft lithography (Figure 13a) [63]. An ID-pattern-engraved PDMS was obtained by hardening the PDMS precursor in a pre-prepared mold. At this time, the electrode finger width, gap, and length of the pattern were 50 μm, 200 μm, and 1 cm, respectively. The PDMS microchannel was covered with another flat PDMS layer using O_2_ plasma treatment, and then the channel was filled with LM. The fabricated LM-based sensor could be stretched up to 100% due to the material characteristics, and the repeatability gauge factor was −0.3.

Meanwhile, Joo et al. (2022) studied the fabrication of capacitive touch sensors using intaglio contact printing, a type of soft lithography technique (Figure 13b) [64]. For this purpose, CNTs were mixed with paraffin to make a slurry in a form suitable for intaglio space on PDMS stamps. The CNT composite filled in the intaglio of the ID pattern was transferred to various substrates and used to produce a capacitive touch sensor. At this time, ID patterns with finger widths of 125, 75, and 50 μm were prepared in the same area, and the number of fingers increased to 24, 40, and 60 as the width decreased. Based on Equation (2), it was shown that resolution and sensitivity increased as the pattern width decreased.

### 3.6. Advantages and Drawbacks

The major advantages and drawbacks of the patterning technologies reviewed in this section (i.e., inkjet printing, screen printing, laser patterning, and soft lithography) are briefly summarized in Table 1.

## 4. 3D Structured Capacitive Sensors

In this section, we introduce 3D structural sensors fabricated using patterning techniques for performance or stability. Conventional 2D structural sensors may exhibit long-term stability and function even when under tens of percent of strain; however, it is difficult to apply them to actual wearable devices [65]. In addition, when they encounter 3D objects, their functioning is very limited because they have extremely localized contact surfaces. To overcome these chronic problems, attempts have been made to produce sensors that deviate from a flat substrate using various techniques such as 3D printing, buckling, origami, and kirigami [66,67,68]. 3D structures are more resistant to external stress and are also useful for increasing the contact area with analytes. Additionally, sensors with a 3D structure can control the strain detection range and improve sensitivity compared to existing 2D sensors. Therefore, 3D structures may be useful for bioelectronics and robotics.

### 4.1. Transformation from 2D to 3D Structures Using Patterning Techniques

**Figure 14:**
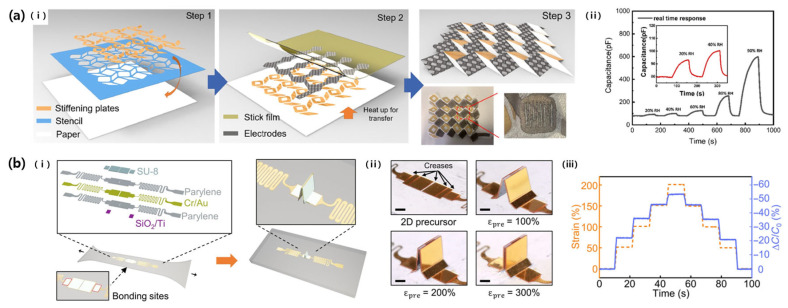
(**a**): (i) Fabrication process of 3D paper-based humidity sensor by using stencil and origami techniques. (ii) Graph of the real-time capacitance change at different humidities. Reproduced with permission from [69], [Applied Materials]; published by American Chemical Society, 2022. (**b**): (i) The structure of the origami inspired strain sensor. (ii) The folding of the sensing area according to the different prestrain. (iii) The graph of the capacitance changes under the strain series of step up and down [70].

In 2022, Chen et al. studied a fiber-attached 3D paper-based origami humidity sensor (POHS) for wearable electronics utilizing the origami technique (Figure 14a) [69]. First, paper substrates were designed into origami tessellation patterns using laser patterning and a stencil technique. Thereafter, a polyester conductive adhesive tape was attached to the substrate via laser patterning. At this time, three patterns were considered: An ID pattern, a non-pattern in a sandwich structure, and a slit pattern in a sandwich structure (the ID-pattern finger width and length were 1 mm and 4.5 mm, respectively, and the slit width was 0.3 mm). The relative change of the measured value for each pattern was 15, 3.2, and 3.6%, respectively, which was the most sensitive in the ID pattern. However, the POHS using the ID pattern was excluded because it was sensitive enough to change the capacitance due to stretching even under constant humidity conditions, and the slit pattern, which had better response and recovery characteristics than the sandwich structure, was chosen. Even though it is a non-stretchable substrate, the humidity sensor obtained elasticity by using an origami pattern and had excellent linearity at a high humidity of 65.3–97.6%. In addition, it had high sensitivity, response and recovery properties, and stability in stretching. However, linearity under low humidity and long-term stability based on the material characteristics were limited.

Huang et al. (2023) also reported an origami-inspired 3D structure strain sensor (Figure 14b) [70]. For this purpose, 2D precursors were fabricated through a mechanically guided assembly process. The sensor consisted of metal electrodes and capping polymers patterned through lithography, in the form of a 5 mm^2^ rectangular sensing area and a serpentine interconnection line. At this time, the partial bonding of the electrode and the foldable crease gives it a unique 3D structure that can be stretched or compressed. Due to its structural advantages, this sensor has a large stretchability of 200%, very low hysteresis (1.2%), a high repeatability rate of more than 700 times (with 100% strain), and a response time of less than 22 ms. The sensitivity increases with designed creases and large prestrain, because of the smaller folding angle of the sensing area, which increases the facing area and makes it closer.

### 4.2. Direct Fabrication of Capacitive Sensors in 3D Structures

**Figure 15:**
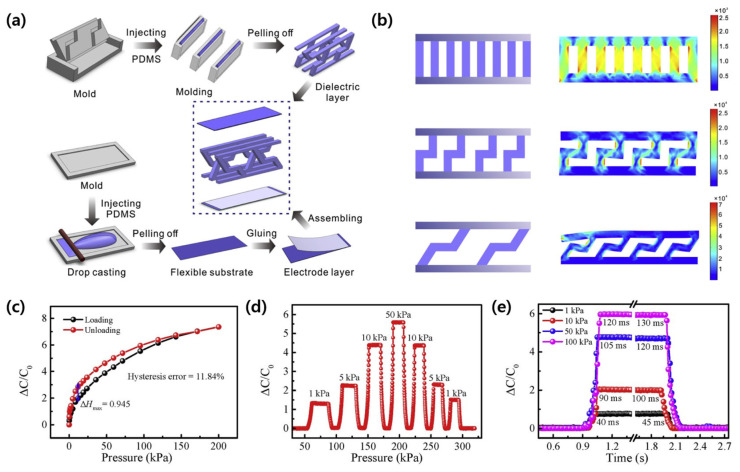
(**a**) The fabrication process schemes of the sensor based on the 3D arrayed frog-leg composite. (**b**) The scheme of different dielectric layer structures and corresponding finite elements modeling under normal pressure. (**c**) The graph showing relative capacitance change with loading and unloading. (**d**) Real-time response to different loading pressure. (**e**) The graph of response and recovery time of the pressure sensor. Reproduced with permission from [71], [Composites Science and Technology]; published by Elsevier, 2023.

Zhao et al. (2023) recently developed a capacitive pressure sensor with a three-dimensional structure by using 3D printing technology to mimic the structure of frog legs (Figure 15) [71]. They created a zigzag mold that mimics frog legs with a 3D printer and then poured PDMS to cure it. The manufactured 3D PDMS was used as a dielectric, and a pressure sensor was fabricated by attaching copper wires to the top and bottom. The sensor based on the 3D arrayed biological frog-leg composites had a LOD of 0.5 Pa, a very high sensitivity of 0.583 kPa^−1^, and a fast response/recovery rate of 40/45 ms.

## 5. Conclusions

This paper reviewed two types of capacitance sensors: The parallel-plate and coplanar types. The parallel-pate type has a very limited application due to its structure, and the most widely used place is the stress sensor. There are many efforts to pattern the surfaces of electrodes and dielectric layers with various forms to improve the performance of stress sensors. On the other hand, the coplanar type is widely used in various fields such as electrochemistry and biotechnology as well as to detect physical movement. Coplanar capacitive sensors are fabricated using various patterning methods, such as inkjet printing, aerosol jet printing, screen printing, laser patterning, and soft lithography. Patterning techniques and patterns directly affect sensor performance, such as resolution and sensitivity, as well as sensor productivity and work processes. Therefore, for this reason, the pattern structure in coplanar sensors is mostly confined to ID patterns. Meanwhile, the performance of sensors can also be improved by establishing a 3D structure through patterning such as 3D printing, buckling, origami, and kirigami. As the structure of the device transformed from 2D to 3D, not only mechanical characteristics but also performance such as resolution and sensitivity could be improved. In summary, sensors will be able to meet the high-performance specifications required by next-generation applications through various patterning technologies.

## Figures and Tables

**Table 1:** Summary of patterning methods with major advantages and drawbacks.

Method	Advantages	Drawbacks	Ref.	Resolution	Applications
Inkjet printing	No mask required	Nozzle clogging	[43,44,45]	300 μm [48]	Wearable tactile sensors
	Large-area printing possible	Low resolution			
	Low cost	Low positioning accuracy			
	High repeatability	Low throughput			
Aerosol jet printing	Non-contact with substrate High resolution No mask required	Low consistency Low reproducibility Cumbersome optimization	[48]	70 µm [53]	Strain sensors
Screen printing	High throughput	Mask required	[64]	570 µm [54]	Wearable pressure sensors
	High precision				
	High resolution				
	Low cost			200 µm [55]	Humidity sensors
	Large-area printing possible				
Laser patterning	No mask required	Damage to materials and substrates	[55,56,65]	800 µm [58]	Microfluidic taste sensors
	High precision				
	Large-area printing possible			200 µm [59]	Biosensors (Thrombin detection)
	High productivity				
	Cost effective			1.5 mm [60]	Humidity sensors
Soft lithography	Simple process	Distortion or damage of elastomeric stamp	[61]	50 µm [63]	Strain sensors
	Low cost	Low repeatability		50 µm [64]	Touch sensors

## Data Availability

Not applicable.
